# Effects of social and environmental restrictions, and changes in alcohol availability in adolescents’ binge drinking during the COVID-19 pandemic

**DOI:** 10.1371/journal.pone.0309320

**Published:** 2024-08-28

**Authors:** Judit Rogés, Marina Bosque-Prous, Cinta Folch, Ester Teixidó-Compañó, Helena González-Casals, Joan Colom, Aina Lafon-Guasch, Paula Fortes-Muñoz, Albert Espelt

**Affiliations:** 1 Facultat de Ciències de la Salut de Manresa, Departament d’Epidemiologia i Metodologia de les Ciències Socials i de la Salut, Research Group in Epidemiology and Public Health in the Digital Health Context (epi4Health), Universitat de Vic-Universitat Central de Catalunya (UVic-UCC), Manresa, Barcelona, Spain; 2 Facultat de Ciències de la Salut, Research Group in Epidemiology and Public Health in the Digital Health context (epi4Health), Universitat Oberta de Catalunya (UOC), Barcelona, Spain; 3 Departament de Psicobiologia i Metodologia de les Ciències de la Salut, Research Group in Epidemiology and Public Health in the Digital Health context (epi4Health), Universitat Autònoma de Barcelona (UAB), Bellaterra, Barcelona, Spain; 4 Centre d’Estudis Epidemiològics sobre les Infeccions de Transmissió Sexual i Sida de Catalunya (CEEISCAT), Agència de Salut Pública de Catalunya, Badalona, Barcelona, Spain; 5 Centro de Investigación Biomédica en Red de Epidemiología y Salud Pública (CIBERESP), Madrid, Spain; 6 Subdirecció General d’Addiccions, VIH, Infeccions de Transmissió Sexual i Hepatitis Víriques, Agència de Salut Pública de Catalunya (ASPCAT), Barcelona, Spain; University of Technology Sydney, AUSTRALIA

## Abstract

**Aims:**

The aim of the present study was to estimate the evolution of binge drinking since the pre-pandemic period, and throughout the pandemic period with the application and lifting of the restrictions in adolescents aged 12 to 19 years old in school in Central Catalonia.

**Methodology:**

Quasi-experimental time series study with two samples of adolescents. The first sample (1^st^ wave of survey, pre-pandemic period) was obtained between the months of September 2019 to March 2020 (n = 6621) and the second sample (2^nd^ wave of survey, pandemic period) between the months of October 2021 and March 2022 (n = 7576). The dependent variable was monthly binge drinking. The main independent variable was the period of data collection (1st and 2nd wave), and gender and grade were also included. Twenty-one time slices were performed by fortnight and the binge drinking prevalence of the previous month was extracted in each of them. Interrupted time series analysis was performed and Poisson regression models with robust variance were estimated.

**Results:**

The data indicated a significant increase in the prevalence of binge drinking in certain periods in girls [easing of measures in October, aPR: 2.25 (1.03–4.89); and total lifting of restrictions in February, aPR: 3.29 (1.57–6.89)] and a reduction in consumption in periods of tightening of restrictions. After the upturn before the return to the pre-pandemic situation binge drinking followed a decreasing trend in both sexes [aPR boys: 0.73 (95%CI: 0.66–0.81); aPR girls: 0.78 (95%CI: 0.71–0.86)].

**Conclusions:**

Periods of community interventions aimed at protecting people’s health have had an impact on other health behaviors or aspects of health such as binge drinking, and differentially across groups and communities.

## Introduction

Alcohol consumption is a public health problem directly and indirectly associated with multiple short- and long-term health problems [1]. Over the years, studies have shown that adolescents are highly vulnerable to the effects of alcohol consumption [2,3]. Among adolescents, alcohol consumption is facilitated by leisure and socialization contexts [4–6], favoring the development of positive aspects such as social recognition, reduced social anxiety, social desirability, fun, and bonding [7]. In addition, poor family control may be a key factor and has been identified as a risk factor for early alcohol consumption [8,9]. In recent years there has been an increase in binge drinking, a practice characterized by the consumption of 6 or more alcoholic beverages in a single drinking occasion or in a period of approximately 2 to 4 hours [10]. According to the latest national surveys, this pattern of consumption is becoming increasingly frequent among young people aged 15 to 24, and in Spain and Catalonia it is estimated that one in four boys and girls have engaged in binge drinking in the last 30 days [2,11,12].

In March 2020 the World Health Organization (WHO) declared a worldwide pandemic situation due to the spread of the SARS-CoV-2 virus (COVID-19). After the containment period, infection data indicated that person-to-person contact was a rapid route of transmission, which is why between September 2021 and February 2022 different universal prevention interventions at the community level were applied in Catalonia, the purpose of which was to curb virus transmission by limiting social contact. Faced with this unknown situation, it was necessary to undertake community actions aimed at prevention and protection of people’s health, taking into account the needs, economic situation, structure and organization of services, and the health status of each community [13]. Interventions were especially focused on nightlife, since these were closed spaces that concentrated large groups of people and where activities such as drinking alcoholic beverages, made it difficult to comply with the health measures in force at the time. In view of this situation, several studies reported a reduction in alcohol consumption in the adolescent population during confinement at the beginning of the COVID-19 pandemic, especially binge drinking [14,15]. This reduction has been associated with the set of interventions implemented in the community, such as the closure of nightlife establishments, and limitations on hours, leisure, and social gatherings. It is also believed that these community interventions have led to a reduction in leisure options, more time spent at home under parental control, and greater difficulty in accessing alcohol [16]. Despite the reduction in alcohol consumption during confinement and restrictive periods, studies have shown an increase in binge drinking and heavy drinking in public spaces [17], and possibly the fact of living in rural areas with less control over mobility facilitated social encounters between adolescents. On the other hand, some studies have indicated slight upturns in binge drinking immediately after the restrictions were lifted, and hypothesize the development of new forms of alcohol consumption among adolescents, with more frequent consumption [18] and a more abusive pattern [18,19].

Some investigations have focused on studying changes in alcohol consumption among the young or adult population during the onset of the pandemic. However, the lack of monitoring studies throughout the entire pandemic period provides little evidence on the effect of community measures or interventions that were implemented after total confinement due to COVID-19. Thus, studies with more regular data collection were needed to compare alcohol consumption before the COVID-19 pandemic and during the different periods of the pandemic, in order to know the impact that community interventions have had in the short term on the health behaviors of the adolescent population, including alcohol consumption. Therefore, the aim of the present study was to estimate the evolution of binge drinking from the pre-pandemic period, and throughout the pandemic period with the application and lifting of the restrictions imposed as preventive measures of community transmission of COVID-19, in a cohort of adolescents aged 12 to 19 years in school in Central Catalonia.

## Material and methods

### Study design and sample

Quasi-experimental time series study with twenty-one time slices over two waves of DESKcohort data collection [20]. The study population was adolescents aged 12 to 19 years old attending school in Central Catalonia who during the 2019–20 and 2021–22 academic years (1^st^ and 2^nd^ waves of the project, respectively) were in the 2^nd^ or 4^th^ year of compulsory secondary education (CSE), 2^nd^ of post-compulsory secondary education (PCSE) or 2^nd^ of Intermediate Level Training Cycles (ILTC). To contact all educational centers in Central Catalonia, at the end of the previous school year’s data collection, we requested the Department of Education of the Government of Catalonia to provide us with the updated list of compulsory secondary and post-compulsory education centers. For the recruitment of the sample, during that time (May-June of the previous school year), we contacted all the educational centers in Central Catalonia to present the project to them and invite them to participate. To the ones agreeing to participate, we also send informed consent forms to be signed by the adolescents’ legal guardians. Then, at the beginning of the academic year in which the data collection will take place (September), we contact the schools to schedule a date for administering the questionnaire. 65 out of 91 educational centers participated in the 1st wave (71.4%), and 84 out of 98 in the second wave (85.7%). As for the participation rate in each region, in the 1st wave, 7,319 adolescents participated out of 11,943 (61.3%), and in the 2nd wave, 9,265 out of 14,342 (64.6%). As for the recruitment period for this study, the 1^st^ wave of the study started on 21^st^ September 2019 and ended on 10^th^ March 2020, referring to the period prior to the COVID-19 pandemic, and a total of 6,621 individuals participated. The 2^nd^ wave started on 30^th^ September 2021 and ended on 6^th^ June 2022, and in this time frame different biweekly periods of post-confinement restrictions followed each other, and in February 2022 all restrictions in place since the beginning of the pandemic were definitively lifted ("new normal", due to the return to the pre-pandemic state). The sample of the second wave included in the study period was 7576 people [20].

For more information on the sample, the percentage of participation, the study variables or the development of the fieldwork during the two waves of the project, see the protocol article translated into English and open access [20].

### Data collection instrument

Data collection was performed using the distribution of DESKcohort questionnaire, which collects data on adolescent health behaviors and their determinants, and on social and contextual aspects. The questionnaire was designed based on other questionnaires to assess health behaviors during adolescence. In the case of alcohol consumption, validated tests and instruments used in other surveys were used [21–25], and have high validity and reliability [26–29].

The questionnaire was self-reported, and data were collected in the classrooms of the participating schools using an electronic device linked to a data storage system (REDcap). The surveys were supervised by project staff specifically prepared for the fieldwork [20].

### Study variables

The main dependent variable was monthly binge drinking, which was collected from the question "*How often do you have 6 or more alcoholic drinks on a single drinking occasion (understanding "occasion" as having drinks in a row or in an approximate interval of 2-4h)*?". The responses of the participants were divided into two categories, with those who indicated that they had binge drinking monthly, weekly or daily or almost daily falling into the "*Monthly binge drinking*" category and the rest into the "*No monthly binge drinking*" category.

The main independent variable was the period in which data were collected in each of the study waves [Wave 1 (Pre-COVID-19); Wave 2 (Restrictive periods)]. In total, twenty-one time slices were performed by fortnight (9 in the 1^st^ wave and 12 in the 2^nd^ wave), and in each of them the age-adjusted prevalence of monthly binge drinking was observed. A fortnightly data collection period was delimited because the interventions applied in leisure and social encounters in Catalonia were reviewed and modified every fortnight by the Generalitat of Catalonia, who dictated the restrictive measures and periods (Resolution SLT/3787/2021) (Table 1) [30]. Other independent variables such as sex, grade, and type of municipality of residence were also included. The variable sex was collected through the survey question "What is your biological sex?", with the response options "Boy" and "Girl". As for grade, participants were directly asked to indicate their current grade level, with the response options "2nd CSE", "4th CSE", "2nd PCSE", and "2nd ILTC". Regarding the variable type of municipality of residence, the survey included a list of towns and cities in each region of the Central Catalonia, and participants were asked to indicate their place of residence. To determine whether the place of residence was considered a rural or urban municipality, updated population census data from the Institut Català d’Estadística (https://www.idescat.cat/) were consulted in each wave of the project for each municipality in the counties included. A dichotomous variable was created for the type of municipality of residence, with categories of "Rural" for municipalities with a population ≤10,000 inhabitants, and "Urban" for municipalities with a population >10,000 inhabitants.

**Table 1:** Interventions to restrict leisure and social contact applied in Catalonia between September 2021 and February 2022, corresponding to the data collection period of the second wave of the project.

Period of time	Leisure	Mobility	Social
**15**^**th**^ **Sept. - 15**^**th**^ **Oct. 2021** *(restrictions)*	Nightclubs closed. Bars and pubs with restrictions on capacity and opening hours.	Without limitations.	Meetings with a maximum of 10 people.
**16**^**th**^ **Oct. - 29**^**th**^ **Oct. 2021** *(restrictions)*	Nightclubs closed.	Without limitations.	Without limitations.
**30**^**th**^ **Oct. - 6**^**th**^ **Nov. 2021** *(restrictions)*	Nightclubs closed. Bars and pubs also closed.	Perimeter confinement. Curfews of the population.	No limitations on meetings.
**7**^**th**^ **Nov. - 22**^**nd**^ **Dec. 2021** *(restrictions)*	Nightclubs closed.	Without limitations.	Without limitations.
**23**^**rd**^ **Dec. 2021 - 28**^**th**^ **Jan. 2022** *(restrictions)*	Nightclubs closed. Bars and pubs also closed.	Perimeter confinement. Curfews of the population.	Meetings with a maximum of 10 people.
**29**^**th**^ **Jan. - 11**^**th**^ **Feb. 2022** *(restrictions)*	Nightclubs and concert halls closed. No limitations in capacity or opening hours for bars and pubs.	Without limitations.	Without limitations.
**12**^**th**^ **Feb. - 11**^**th**^ **Mar. 2022** *(no restrictions)*	Total reopening of discotheques, bars and pubs (return to pre-pandemic situation).	Without limitations.	Without limitations.

### Analysis plan

First, the baseline characteristics of the 1^st^ and 2^nd^ wave sample were described. Then, for each fortnight, we calculated the monthly binge drinking prevalence adjusted for age and type of municipality, with a 95% confidence interval (95%CI) and compared the prevalences over the time series of the different fortnight periods of the 1^st^ wave (pre-COVID-19 time) to the same fortnights during the 2^nd^ wave (restrictive periods). The temporal evolution of age and municipality type adjusted prevalences of binge drinking throughout the study period was plotted in a time series graph for both sexes separately. To assess changes in the age and municipality type adjusted prevalence of monthly binge drinking after each period with restrictions during the 2nd wave, an interrupted time series analysis was performed. The model collects data from the following formula:

$$\begin{array}{l} \ln[E(Y_{t})]=\beta_{0}+\beta_{1}T_{t}+\beta_{2}X_{t}\\ \;+\sum_{k}[\beta_{3k}\sin(\frac{2K\pi }{T})+\beta_{4k}\cos(\frac{2K\pi }{T})]+\beta_{5}X_{t}T_{t}\\ \;+\sum_{j}(\beta_{6j}Z_{jt}) \end{array}$$

**Fig 1:**
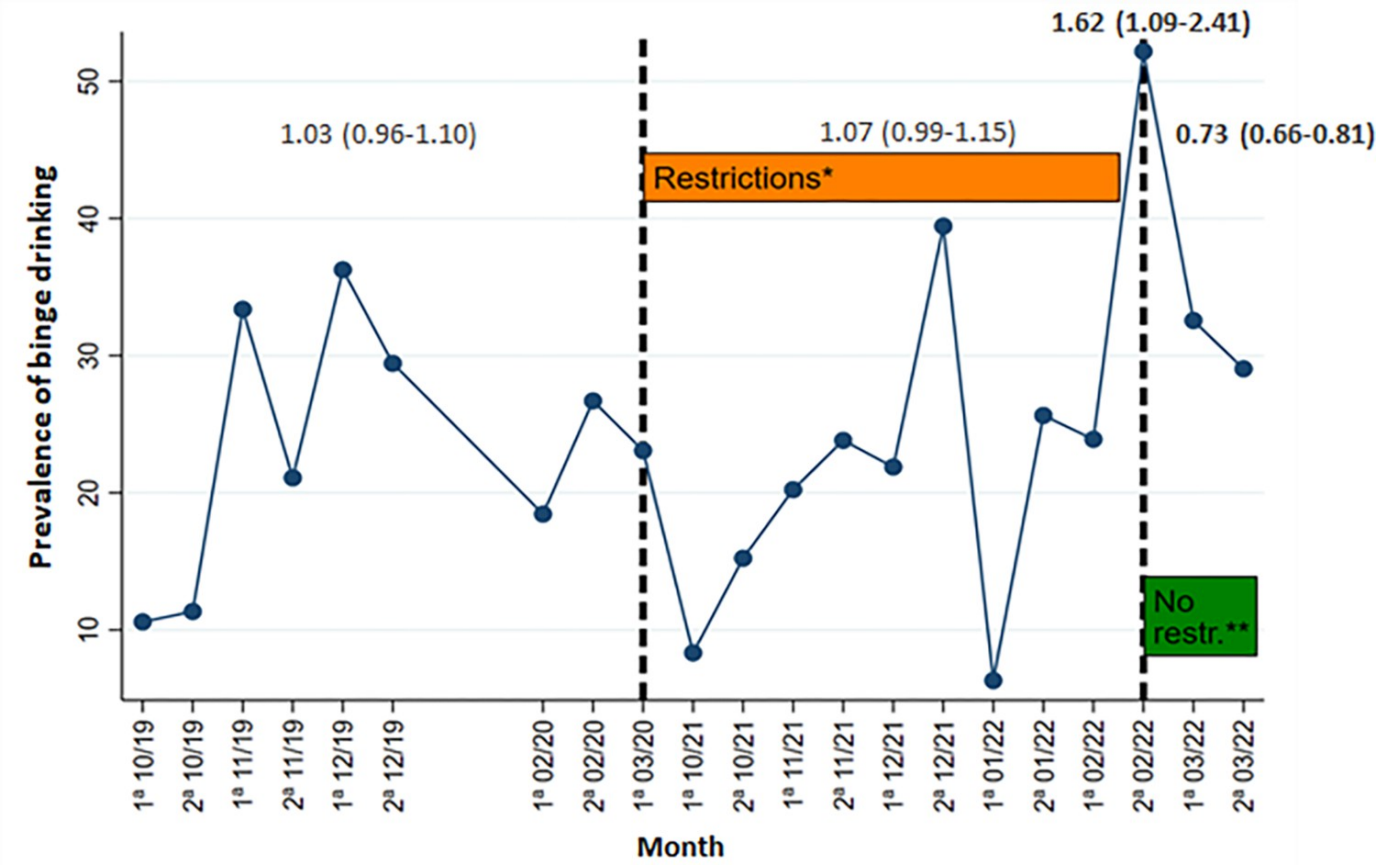
Line graph of binge drinking prevalence over the 2019–2022 time series, for boys. *Restrictions: Total closure of discotheques, curfew periods for the population, limitation of indoor capacity, and limitation of social gatherings (max. 10 people). **No restrictions: Total reopening of discotheques, bars and pubs, return of nightlife and socializing to pre-pandemic situation.

**Fig 2:**
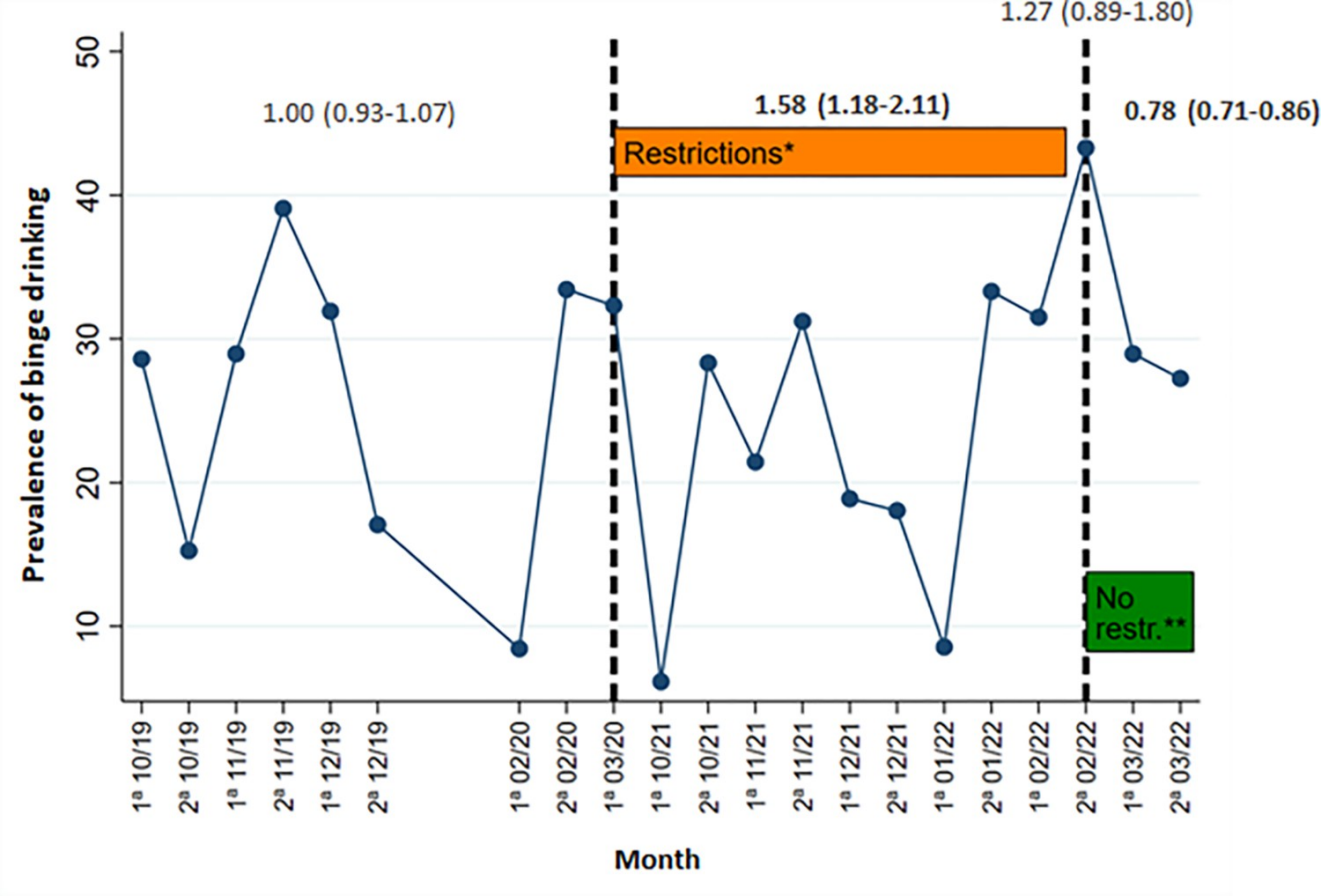
Line graph of binge drinking prevalence over the 2019–2022 time series, for girls. *Restrictions: Total closure of discotheques, curfew periods for the population, limitation of indoor capacity, and limitation of social gatherings (max. 10 people). **No restrictions: Total reopening of discotheques, bars and pubs, return of nightlife and socializing to pre-pandemic situation.

Where Y_t_ is the monthly binge drinking prevalence adjusted for academic year and municipality of residence recorded at time t (t = 1,…, T). T_t_ is the time period (T_1_ = 1 for the first fortnight, T_2_ = 2 for the second one, etc.); X_t_ identifies the pre-constraint (X_t_ = 0) and post-constraint (X_t_ = 1) period of each constraint period; K takes values between 1 and 6 (K = 1 for annual seasonality; K = 2 for semiannual seasonality, etc.); T is the number of periods described by each sinusoidal function (e.g. T = 21 fortnights); Z_jt_ are other covariates introduced; and j is the number of covariates introduced. The time series model was fitted using only a sinusoidal function (Figs 1 and 2) with age-adjusted prevalence. The age-adjusted prevalences of binge drinking for each period were adjusted using the entire population and the direct method [31]. Subsequently, in Tables 3 and 4, Poisson regression models with robust variance were employed to obtain the relative differences in the Prevalence of each period. Prevalence Ratios, with their corresponding 95% confidence intervals, were adjusted for age and municipality type (adjusted Prevalence Ratios, aPR), as previously described [32]. Data analysis was conducted using the statistical software STATA 17.

### Ethical considerations

The study was approved by the Ethics Committee of the Universitat de Vic-Universitat Central de Catalunya (UVic-UCC) (96/2019). Participation in the study was voluntary. The criteria required for participation were to be between 12 and 19 years of age, and to have signed the written informed consent (in the case of persons under 14 years of age signed by a family member or legal guardian). The same Ethics Committee approved the informed consent document, and this was sent to all educational centers prior to the administration of the survey. On the day of the survey, the researchers collected the consents signed by the parents or legal guardians of the participating adolescents, in written format.

## Results

Table 2 shows the characteristics of the participants in the two waves of the DESKcohort project. A total of 6621 people (52.4% girls) participated in the 1^st^ wave and 7576 (51.3% girls) in the 2^nd^ wave. Compared to the 1^st^ wave, in the 2nd wave participation decreased in 2^nd^ year of CSE (2.3%) and increased slightly in 4^th^ year of CSE (0.8%), 2^nd^ year of PCSE (1.3%) and 2^nd^ year of ILTC (0.2%), the latter being the academic course with the lowest participation in the study. Overall, 7.6% of people in the 1^st^ wave and 9.0% in the 2^nd^ wave reported doing binge drinking in the previous month.

**Table 2:** Description of the sample of the 1^st^ and 2^nd^ waves of the study.

	1^st^ Wave (pre-COVID-19)		2^nd^ Wave (restrictive periods)		
	N	%	N	%	p-value
	6621	100	7576	100	
***Sex***					0.169
Boys	3150	47.6	3884	48.7	
Girls	3471	52.4	3692	51.3	
***Grade***					0.034
2^nd^ CSE	2262	34.2	2418	31.9	
4^th^ CSE	2444	36.9	2859	37.7	
2^nd^ PCSE	1476	22.3	1785	23.6	
2^nd^ ILTC	439	6.6	514	6.8	
***Data collection fortnight***					<0.001
1^st^ half of October	590	8.9	269	3.6	
2^nd^ half of October	590	8.9	558	7.4	
1^st^ half of November	885	13.4	764	10.1	
2^nd^ half of November	1042	15.7	974	12.9	
1^st^ half of December	868	13.1	505	6.7	
2^nd^ half of December	858	13.0	675	8.9	
1^st^ half of January*	-	-	310	4.1	
2^nd^ half of January*	-	-	591	7.8	
1^st^ half of February	576	8.7	707	9.3	
2^nd^ half of February	715	10.8	1061	14.0	
1^st^ half of March	496	7.5	559	7.4	
2^nd^ half of March*	-	-	603	8.0	
***Binge drinking in the last month***					0.002
No	6120	92.4	6894	91.0	
Yes	501	7.6	682	9.0	

* In the 1^st^ wave of the DESKcohort project, no surveys were administered during the month of January or the second half of March, so no data were available for that period.

Figs 1 and 2 show that in the 1^st^ wave (October 2019-March 2020) binge drinking in the previous month oscillated between 10.6% and 36.3% in boys and 8.4% and 39.1% in girls, with an increase in prevalence during the month of November (33.4% boys; 39.1% girls). In the 2^nd^ wave, we also observed an increase in November 2021. This rebound coincides with the end of a period of social and mobility restrictions, which ran from September 15 to October 15. After this increase, data show a subsequent reduction of the binge drinking in December 2021 (29.4% boys; 17.1% girls), coinciding with a period of severe restrictions due to the resurgence of COVID-19 cases, which involved the closure of nightclubs, perimeter confinements and limitations on social gatherings. Compared to the 1^st^ wave, during the restriction period (October 2021-February 2022) prevalences among boys increased progressively between October and December, coinciding with periods of few social restrictions, consumption peaks were observed, while instances of binge drinking declined during the implementation of restrictive measures. From January to 1^st^ fortnight of February more variability in the data was observed (minimum prevalence of 6.3% and maximum of 52.2%), because in January was the month with the most severe restrictions throughout the study period, and in February all of them were removed. In the case of girls, prevalences during the 2^nd^ wave were more variable compared to the 1^st^ wave, with values ranging from 6.5% to 43.3%. After the removal of community interventions in the 2^nd^ fortnight of February 2022, a spike in the prevalence of binge drinking was observed in boys (52.2%) and an increase in girls (43.3%), which was not observed in the same month in the 1^st^ wave. These prevalences were the highest recorded over the entire study period. In the weeks following this spike, prevalences decreased for both sexes, stabilizing at values similar to the pre-COVID19 period.

Tables 3 and 4 allow to assess the comparability of samples over time, providing data about the characteristics of the sample in each fortnight.

**Table 3:** Description of the characteristics of the sample of the 1^st^ wave of the study according to each fortnightly period of the time series.

	1^st^ Wave (pre-COVID-19)											
	October 2019		November 2019		December 2019		January 2020		February 2020		March 2020	
	1^st^ half	2^nd^ half	1^st^ half	2^nd^ half	1^st^ half	2^nd^ half	1^st^ half	2^nd^ half	1^st^ half	2^nd^ half	1^st^ half	2^nd^ half
	%	%	%	%	%	%	%	%	%	%	%	%
***Sex***												
Boys	48.3	49.3	45.3	48.6	48.0	47.2	0.0	0.0	48.1	46.9	46.4	0.0
Girls	51.7	50.7	54.7	51.4	52.0	52.8	0.0	0.0	51.9	53.1	53.6	0.0
***Grade***												
2^nd^ CSE	24.1	48.3	24.3	32.4	26.4	43.8	0.0	0.0	38.2	32.6	45.8	0.0
4^th^ CSE	45.6	26.2	39.5	45.2	28.6	38.6	0.0	0.0	37.8	30.9	36.3	0.0
2^nd^ PCSE	30.3	6.0	30.8	15.7	30.2	14.1	0.0	0.0	24.0	29.9	17.9	0.0
2^nd^ ILTC	0.0	19.5	5.3	6.6	14.9	3.5	0.0	0.0	0.0	6.6	0.0	0.0
***Municipality of residence***												
Rural	34.5	46.0	57.0	58.3	35.6	64.4	0.0	0.0	65.5	58.1	64.1	0.0
Urban	65.5	54.0	43.0	41.7	64.4	35.6	0.0	0.0	34.5	41.9	35.9	0.0
***Binge drinking in the last month***												
No	91.2	94.0	91.3	93.4	89.9	95.2	0.0	0.0	95.0	90.6	91.1	0.0
Yes	8.8	6.0	8.7	6.6	10.1	4.8	0.0	0.0	5.0	9.4	8.9	0.0

**Table 4:** Description of the characteristics of the sample of the 2^nd^ wave of the study according to each fortnightly period of the time series.

	2^nd^ Wave (restrictive periods)											
	October 2021		November 2021		December 2021		January 2022		February 2022		March 2022	
	1^st^ half	2^nd^ half	1^st^ half	2^nd^ half	1^st^ half	2^nd^ half	1^st^ half	2^nd^ half	1^st^ half	2^nd^ half	1^st^ half	2^nd^ half
	%	%	%	%	%	%	%	%	%	%	%	%
***Sex***												
Boys	47.2	45.2	46.5	48.7	47.1	48.9	46.5	48.6	52.2	51.2	50.3	48.4
Girls	52.8	54.8	53.5	51.3	52.9	51.1	53.5	51.4	47.8	48.8	49.7	51.6
***Grade***												
2^nd^ CSE	57.2	33.3	41.5	38.7	38.2	33.9	45.2	25.7	18.0	18.7	22.4	36.5
4^th^ CSE	18.6	44.8	42.1	42.2	41.2	32.6	43.2	45.5	27.9	40.4	28.4	34.8
2^nd^ PCSE	24.2	21.9	13.6	15.2	20.6	26.2	11.6	21.0	27.4	33.6	32.6	28.7
2^nd^ ILTC	0.0	0.0	2.7	3.9	0.0	7.3	0.0	7.8	26.7	7.4	16.6	0.0
***Municipality of residence***												
Rural	54.9	64.7	93.1	56.9	57.4	31.9	61.6	25.1	63.4	27.0	70.4	29.4
Urban	45.1	35.3	6.9	43.1	42.6	68.1	38.4	74.9	36.6	73.0	29.6	70.6
***Binge drinking in the last month***												
No	94.4	90.5	92.5	94.0	92.1	92.3	93.5	90.5	87.7	89.2	87.3	90.4
Yes	5.6	9.5	7.5	6.0	7.9	7.7	6.5	9.5	12.3	10.8	12.7	9.6

On the other hand, binge drinking PRs were calculated for each fortnight throughout the period of restrictions for boys and girls, adjusted for the variables course and municipality of residence (aPR). The results are presented in Table 5, which includes the aPR with their respective 95%CIs. The prevalence of binge drinking increased significantly among girls in the second half of October [aPR: 2.25 (1.03–4.89)] with respect to previous months, matching with the softening of the measures in force during the summer period. Despite the absence of statistical significance, the data suggest a reduction in the prevalence of binge drinking in the face of intensified restrictions in the month of November of the 2^nd^ wave and an increase in boys between the second fortnight of November [aPR: 1.12 (0.67–1.85)] and the second fortnight of December 2021 [aPR: 1.28 (0.79–2.08)], matching with the removal of these measures. Compared to the respective fortnights of the 1^st^ wave, it is observed that, with the easing of restrictions at the beginning of February 2022, the prevalence of binge drinking was higher among girls [aPR: 3.29 (1.57–6.89)]. With respect to the 1^st^ wave, with the lifting of restrictions and the return to the pre-pandemic state in mid-February 2022, no statistically significant differences in binge drinking were observed.

**Table 5:** Adjusted Prevalence Ratios (aPR) of binge drinking for each fortnight during the pre-COVID19 (1^st^ Wave 2019–2020) and post-COVID19 (2^nd^ Wave 2021–22) period, for boys and girls.

	aPR			
	Boys		Girls	
	Pre-COVID-19	Post-COVID-19	Pre-COVID-19	Post-COVID-19
1^st^ half of October*	1.00	1.78 (0.77–4.11)	1.00	0.58 (0.26–1.32)
2^nd^ half of October**	1.00	1.17 (0.52–2.63)	1.00	**2.25 (1.03–4.89)**
1^st^ half of November***	1.00	0.98 (0.62–1.56)	1.00	1.32 (0.85–2.06)
2^nd^ half of November**	1.00	1.12 (0.67–1.85)	1.00	0.99 (0.64–1.53)
1^st^ half of December**	1.00	1.41 (0.84–2.37)	1.00	1.02 (0.57–1.83)
2^nd^ half of December***	1.00	1.28 (0.79–2.08)	1.00	1.06 (0.54–2.06)
1^st^ half of February***	1.00	0.93 (0.50–1.71)	1.00	**3.29 (1.57–6.89)**
2^nd^ half of February**	1.00	1.04 (0.70–1.52)	1.00	0.94 (0.62–1.41)
1^st^ half of March**	1.00	1.02 (0.60–1.74)	1.00	0.76 (0.42–1.38)

In the 1st wave of the DESKcohort project, no surveys were administered during the month of January or the second half of March, so no data are available for that period. Model adjusted by grade and municipality of residence.

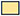
*Partial restrictions on leisure and social gatherings.

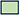
**Mild restrictions on social gatherings and closed nighttime leisure.

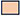
***Total restrictions on leisure and social gatherings.

We also analyzed the PRs of the changes in binge drinking adjusted by age and municipality of residence and the trend over the three study periods (Pre-COVID19; With restrictions; Without restrictions). The results are presented in Table 6, where the comparison between the 1^st^ wave and the 2^nd^ wave of the study can be observed. Compared to the pre-COVID19 period, boys had a significantly higher prevalence of binge drinking after the removal of restrictions in wave 2 [aPR: 1.62 (1.09–2.41)]. A higher prevalence of binge drinking was also observed in girls at wave 2 compared to wave 1, but these differences were not statistically significant [aPR: 1.27 (0.89–1.80)]. On the other hand, the prevalence of binge drinking among girls followed an increasing trend during the restrictions period of the 2^nd^ wave [aPR: 1.58 (1.18–2.11)] with respect to the pre-COVID19 period of the 1^st^ wave. Finally, after removal of the restrictions and return to the pre-COVID19 situation, the results showed a decreasing trend and subsequent stabilization of binge drinking compared to the pre-COVID19 period for both boys [aPR: 0.73 (0.66–0.81)] and girls [aPR: 0.78 (0.71–0.86)].

**Table 6:** Adjusted Prevalence Ratios (aPR) of changes in the prevalence of binge drinking in the previous month and trend values, for each study period according to sex.

	Boys	Girls
	aPR (95%CI)	aPR (95%CI)
***Absolute change***		
Pre-COVID19 (1^st^ Wave)	1	1
***Restrictions*** (2^nd^ Wave)	0.88 (0.59–1.30)	0.84 (0.58–1.22)
No restrictions (2^nd^ Wave)	**1.62 (1.09–2.41)**	1.27 (0.89–1.80)
***Trend***		
Pre-COVID19 (1^st^ Wave)	1.03 (0.96–1.10)	1.00 (0.93–1.07)
Restrictions (2^nd^ Wave)	1.07 (0.99–1.15)	**1.58 (1.18–2.11)**
No restrictions (2^nd^ Wave)	**0.73 (0.66–0.81)**	**0.78 (0.71–0.86)**

Model adjusted by course and municipality of residence.

## Discussion

To date, there have been numerous studies on the impact of COVID-19 measures on drinking patterns comparing pre- and post-pandemic periods, and few studies have accounted for the seasonality of the restrictions imposed over time. Therefore, the data from the present study allow us to observe the evolution of monthly binge drinking throughout the pandemic and show differences between the period before and during COVID-19, and a seasonal change in binge drinking related to the establishment or suppression of restrictions in the community.

During adolescence, alcohol consumption is facilitated by socialization contexts and being with peers [4–6]. Especially the abusive consumption of alcohol is encouraged in establishments related to leisure, and to a greater extent in nightlife establishments such as bars or nightclubs. Community intervention actions during the pandemic were implemented with the intention of preventing the spread of the virus among the population, and consequently affected the routine, lifestyles and health behaviors of young people, such as alcohol consumption [33]. First, the results of the present study suggest that in the period prior to COVID-19, binge drinking remained stable among adolescents. Secondly, that during the pandemic period marked by community interventions, consumption evolved differently between groups, with a tendency towards a reduction among boys and an increase among girls. Finally, when the limitations were removed and leisure and social contact were adapted to pre-COVID-19 conditions, there was an initial tendency for consumption to increase, and a subsequent reduction and stabilization of consumption. The fact that in the study sample there was a high prevalence of young people who were in their 2^nd^ year of CSE could explain the slight reduction in consumption during the periods of restrictions among boys, maybe due to the increase in the difficult of accessibility to alcohol. It is hypothesized that these people were already binge drinking prior to the periods of restrictions, and had fewer difficulties in acquiring alcohol and maintaining this pattern of use despite the limitations, so that their consumption during the pandemic did not vary with respect to the pre-COVID-19 period [17,32]. In the case of girls, possibly before the pandemic, their consumption was lower and associated with entertainment and nightlife [33], and the fact of having lived through restrictive times increased their motivation to consume more intensively in less controlled environments [34]. In fact, the results were similar to another study with a Norwegian population, which suggests that despite the regulations applied, adolescents did not necessarily have difficulties in accessing and maintaining their consumption of alcohol [18]. Likewise, other studies have found various explanations, such as that young people found alternative spaces to leisure establishments in which to consume (their own home or home of someone close by), and diversified their consumption channels, drinking during video calls or other remote means that they had frequented during the total confinement by COVID-19 [35,36].

On the other hand, the results show that, in general, prevalence of binge drinking varies according to the type of restrictions that were implemented. In this line, slight reductions in consumption were observed when community restrictions were imposed, and an increase in consumption after their relaxation. In the case of the present study, this is clearly seen in the time frame of the second half of December 2021, where there is a peak in consumption among boys possibly associated with the absence of restrictions and a festive period. As stated by other authors, when a restrictive period is lifted, alcohol consumption increases for a short period of time, justified by the return to social life and the need to make up for lost time [19]. On the contrary, in October 2021 and January 2022, the lowest consumption of the entire study period is recorded. Possibly during these periods, severe restrictions on nightlife, mobility, and socialization reduced the availability of alcohol and decreased opportunities for consumption among adolescents [37]. At the end of January 2022 the government began to reduce the limitations, and it is from then on, that an increasing trend of binge drinking was observed among the adolescent population. In February 2022, with the removal of all current restrictions and the reopening of nightlife after two years of closure, the highest prevalence of binge drinking of the entire study period was observed in boys, and an upturn compared to previous months in girls [38]. The upturn in binge drinking in the face of the removal of restrictions makes plausible the relationship between leisure, social life and alcohol consumption in adolescents, so that the limitation in the former is associated with a reduction in the accessibility of alcohol and a change in drinking patterns, closely related to social activities [33,39].

The present study shows differences in the amount and pattern of alcohol consumption according to sex. Some authors point out that boys drink more when they are exposed to stress and that girls prefer to drink in situations of relaxation and entertainment [33]. However, the results of the present study coincide with other research and show a higher tendency of binge drinking during the restriction period among girls compared to boys [34]. The literature reports that girls are more likely than boys to access alcohol through their peers, so a reduction in binge drinking was expected in the face of limited social encounters [37]. Despite the contrariness of the results of the present study, it is hypothesized that the restrictions did not hinder accessibility to alcohol among adolescents, who were able to consume at home with the family, or who found alternative environments to entertainment establishments in which to consume high quantities of alcohol [35].

On the other hand, festive periods are times of greater exposure to alcohol and consumption at home, and where leisure activities with peers and celebrations increase. This pattern is evident throughout the study, since in both the 1^st^ and 2^nd^ waves there were increases in binge drinking during Christmas and local holiday periods (December), and a subsequent decrease and stabilization of consumption before the return to daily activities. These data suggest that, even with the application of restrictive community interventions, adolescents found spaces to meet with their peers and consume alcohol, or even that family gatherings were facilitating contexts for drinking [17,35].

In short, community interventions have generated changes in health behaviors, such as alcohol consumption among adolescents, who during the periods of restrictions have been evoked to develop new consumption patterns adapted to the social and health casuistry applied in the community environment.

## Limitations

Finally, it is important to highlight several limitations that should be mentioned. First, the DESKcohort project excludes the population outside the formal educational system; therefore, the sample is not representative of all adolescents aged 12 to 19 years. In addition, people outside the formal educational system could be those who present greater social problems and risky health-related behaviors, for example, greater alcohol abuse, alcohol consumption, and alcoholism [40]. Likewise, the situation of COVID-19 during the 2^nd^ wave of fieldwork (academic year 2021–22) and the health indications for action in the educational setting meant that in some cases at the time of the survey there were students who were confined or absent, which generated losses in participation. Nevertheless, participation was high (64.6%), which has allowed us to have robust and representative data. On the other hand, the DESKcohort questionnaire was self-reported, which could have generated responses subject to biases such as, for example, social desirability or recall. However, the evidence affirms that the use of self-reported questionnaires is a reliable method for detecting alcohol consumption in adolescents [41,42]. On the other hand, the variables sex and academic year were not controlled for the respondents in the different periods of the 1st and 2nd waves, so that the number of participants from each year and educational center could have been different in each wave. Nevertheless, it is considered that the sample follows a normal distribution and that it was possible to counteract the possible effects of this lack of matching, such as differences between sexes attributed to a greater number of boys or girls surveyed in a given period. Another limitation is that the data analysis could not take into account the severity of the restrictions, since the severity of the restrictions varied according to the municipality and was adapted to factors such as the size of the municipality and the health situation at the time.

## Conclusions

The present study provides interesting data on how the interventions applied with the aim of preventing the increase in COVID-19 infections and protecting people’s health have had a differential impact on health behaviors or aspects of health among groups and communities. The measures imposed reduced leisure activities and possibly led young people to opt for other consumption alternatives. The restrictions also made it more difficult to purchase and consume alcohol and led to a change in the consumption trend among the adolescent population.

The seasonal changes observed show the influence of the personal and social context on alcohol consumption among young people. In this line, it is important to plan community interventions with their direct and indirect impact in mind, and to evaluate them taking into account the multiplicity of differential effects, due to gender, socioeconomic, environmental or cultural aspects, that they can have on individuals or groups in the same community.

In addition to assessing the impact of community interventions, the study could be an indicator when designing programs aimed at reducing alcohol consumption among the adolescent population. Knowing how environmental and social measures aimed at minimizing health problems influence on health-related behaviors of adolescents, such as alcohol consumption, provides useful data for public authorities to adapt preventive and health promotion activities to better meet the needs of young people. The findings are conclusive when it comes to seeing the need for this type of policies and programs to modify behaviors of the population that negatively influence their health, especially in crisis situations where public health has a very relevant role in the prevention of health and the well-being of the population. For example, municipal regulations could be proposed to limit the opening hours of nightlife establishments or to further control the accessibility and purchase of alcohol. Likewise, since alcohol use among young people is considered a public health problem, the immediate changes observed in consumption patterns when aspects of the environment are modified demonstrate the importance of transforming environments into healthy spaces. Prevention programs could be designed to offer healthy leisure alternatives aimed at this target population. Sensitization sessions could also be carried out with families and community health agents to raise awareness of the modeling of their behavior and the presence of alcohol in homes or public spaces in the behavior of adolescents. In addition to prevention actions, the results would be used to carry out promotion actions related to the creation of healthy public spaces that favor healthy behaviors for the adolescent and young population. Finally, it would be interesting to disseminate these results to the population to raise awareness of the high prevalence of alcohol abuse and the need for an environment with health-generating resources. Finally, as a proposal for the future, it would be interesting to monitor and carry out a follow-up study with adolescents to find out how the trend of binge drinking evolves over time and to analyze consumption patterns in the post-COVID-19 period.
