# Correction: Effects of social and environmental restrictions, and changes in alcohol availability in adolescents’ binge drinking during the COVID-19 pandemic

**DOI:** 10.1371/journal.pone.0329652

**Published:** 2025-08-04

**Authors:** Judit Rogés, Marina Bosque-Prous, Cinta Folch, Ester Teixidó-Compañó, Helena González-Casals, Joan Colom, Aina Lafon-Guasch, Paula Fortes-Muñoz, Albert Espelt

The following information is missing from the Acknowledgement statement: This article is part of the doctoral thesis of Judit Rogés at the Universitat Oberta de Catalunya.
